# The application of 6S in the care of COVID-19 patients: a Japanese perspective

**DOI:** 10.1186/s13054-020-03261-2

**Published:** 2020-09-01

**Authors:** Mitsuhito Soh, Toru Hifumi, Shutaro Isokawa, Norio Otani, Shinichi Ishimatsu

**Affiliations:** Department of Emergency and Critical Care Medicine, St. Luke’s International Hospital, 9-1 Akashi-cho, Chuo-ku, Tokyo, 104-8560 Japan

**Keywords:** COVID-19, 5S, Shitsuke

Dear Sir:

Wei et al. introduced the original 5S and transformed 6S into 6S management mode for application in the nursing of coronavirus disease 2019 (COVID-19) patients [1]. As the authors described, the 5S concept is comprised of five Japanese words (Seiri, Seiton, Seiso, Seiketsu, and Shitsuke). 5S is among the Japanese customs that have been taught by parents since childhood in Japan and passed down the generations. Therefore, we are familiar with those words, and we would like to comment on the following three points.

First, the authors transformed five Japanese words into the 6S management strategy for convenience of application and understanding in the nursing of COVID-19 patients. However, there are two incorrect descriptions in Figure 1. The fourth S should not be Seiton but Seiketsu, and the fifth should not be Seiketsu but Shitsuke. “Seiketsu” means to continuously revise and maintain the results of Seiri, Seiton, and Seiso.

Second, the authors interpreted “Shitsuke” as “specialization and cooperation.” However, we believe “sustain rules and values” is a much closer translation of “shitsuke.” “Shitsuke” means to make someone not only follow the rules strictly, but also take over the good sense of value (originally, from parents to children) and cooperation with others with it. This sense of value is applied in the nursing management of COVID-19 patients as follows: In the high-stress COVID-19 work environment as the nurse, a good sense of value reduces further stress, sustains compassion for the patients, and facilitates cooperation within the nursing team.

Third, 5S, overall, means environmental (physical) cleanliness, which, indirectly, influences cleanliness in the mind. Cleanliness is similar to purity, which is discussed in Bushi-do, a traditional Japanese code of conduct [2]. This cleanliness in the mind is closely associated with good behavior, not only in the Japanese, but in all other peoples and situational contexts. As an example, the crime rate in New York declined predominantly when Mayor Giuliani eliminated graffiti in downtown New York [3].

Thus, the 5S or 6S concept is very valuable not only in the hospital setting but also in spiritual training as a person. We believe that this spiritual training would contribute to a positive impact on healthcare workers during the COVID-19 pandemic.

## Data Availability

Not applicable

## Abbreviation

COVID-19: Coronavirus disease 2019
